# Pilot Test of Dosage Effects in HEXORR II for Robotic Hand Movement Therapy in Individuals With Chronic Stroke

**DOI:** 10.3389/fresc.2021.728753

**Published:** 2021-10-01

**Authors:** Ji Chen, Iian Black, Diane Nichols, Tianyao Chen, Melissa Sandison, Rafael Casas, Peter S. Lum

**Affiliations:** ^1^Department of Mechanical Engineering, University of the District of Columbia, Washington, DC, United States; ^2^Department of Biomedical Engineering, The Catholic University of America, Washington, DC, United States; ^3^MedStar National Rehabilitation Network, Washington, DC, United States; ^4^Biomedical Engineering Department, Florida International University, Miami, FL, United States

**Keywords:** upper extremity, stroke, neurorehabilitation, robotics, movement disorders, trial registration

## Abstract

Impaired use of the hand in functional tasks remains difficult to overcome in many individuals after a stroke. This often leads to compensation strategies using the less-affected limb, which allows for independence in some aspects of daily activities. However, recovery of hand function remains an important therapeutic goal of many individuals, and is often resistant to conventional therapies. In prior work, we developed HEXORR I, a robotic device that allows practice of finger and thumb movements with robotic assistance. In this study, we describe modifications to the device, now called HEXORR II, and a clinical trial in individuals with chronic stroke. Fifteen individuals with a diagnosis of chronic stroke were randomized to 12 or 24 sessions of robotic therapy. The sessions involved playing several video games using thumb and finger movement. The robot applied assistance to extension movement that was adapted based on task performance. Clinical and motion capture evaluations were performed before and after training and again at a 6-month followup. Fourteen individuals completed the protocol. Fugl-Meyer scores improved significantly at the 6 month time point compared to baseline, indicating reductions in upper extremity impairment. Flexor hypertonia (Modified Ashworth Scale) also decreased significantly due to the intervention. Motion capture found increased finger range of motion and extension ability after the intervention that continued to improve during the followup period. However, there was no change in a functional measure (Action Research Arm Test). At the followup, the high dose group had significant gains in hand displacement during a forward reach task. There were no other significant differences between groups. Future work with HEXORR II should focus on integrating it with functional task practice and incorporating grip and squeezing tasks.

**Trial Registration:** ClinicalTrials.gov, NCT04536987. Registered 3 September 2020 - Retrospectively registered, https://clinicaltrials.gov/ct2/show/record/NCT04536987.

## Introduction

There are 795,000 new strokes in the U.S. each year, and there are currently 7.2 million adult Americans living with stroke (1). The associated costs are 40.1 billion annually. After the acute and subacute recovery phases, individuals with stroke move into the chronic phase (>6 months post) where they often need continued rehabilitation, on-going care and emotional support (2). There's increasing evidence that rehabilitation in this chronic phase can impact quality of life (3). In many cases, individuals regain skills and return to independent living. However, many do not receive the appropriate amount of rehabilitation therapies needed to maximize recovery due to constraints of the current health care system related to rehabilitation services (4).

In the upper extremity, reaching and grasping movements are often impaired and a focus of rehabilitation (5, 6). At 3 months post stroke, hand impairments are the most commonly reported impairment after stroke (7). Typical impairments are hypertonia (increased passive resistance to movement), inability to activate extensors, and abnormal co-contraction of flexors (8). Interjoint coordination and modulation of activation patterns can also be impaired (9). Hand rehabilitation remains very difficult as control of many joints and muscle groups is required to produce a coordinated grasp. Movement therapies include stretching to reduce flexor hypertonia and prevent contractures, and practice of grasp and release tasks in different arm postures. However, repetitive practice of grasping tasks is difficult and frustrating for patients with moderate-severe impairments. Technologies, such as robotics, provide assistance *via* forces applied to the limb that may facilitate more effective practice, allowing completion of movements that would otherwise be impossible. A large body of work now exists in the area of robotic therapy. A recent meta-analysis of 45 studies including 1,619 individuals with stroke, reported robotic therapy improved Activities of Daily Living (ADL) ability, function and muscle strength; however it's unclear what fraction of individuals will achieve long-term clinically meaningful gains (10).

Hand therapy robots can be divided into devices designed to be worn and used as part of ADL or devices that focus on hand movement isolated from the proximal arm (11). Each approach has advantages and disadvantages. Wearable devices can be used during whole upper extremity tasks, such as reach and grasp tasks, and can take the form of active (12–20) or passive exoskeletons (21–24), with a growing emphasis on soft robotics (25). However, because of space and weight constraints in wearable devices, movement kinematics and control algorithms can often be more precise and sophisticated with desktop devices that isolate finger movements, but don't allow use of the hand with objects or in conjunction with proximal arm joints (26–33).

The hand robotic devices that have been tested clinically are showing promising results. For example, the X-Glove is a portable device with 5 linear actuators that independently extend the digits (34). A clinical trial using the X-Glove in subacute stroke showed significant gains in clinical scales of impairment and function after 15 treatment sessions of 30 min of passive stretching followed by active-assisted, task-oriented training. Another clinical trial from this group in chronic stroke with the VAEDA glove using voice and EMG-control showed advantages in functional scales compared with control therapy without the glove (35). Amadeo (Tyromotion, Austria) is a tabletop hand robot that provides independent motion of all five fingertips along linear paths. A pilot clinical trial using Amadeo in chronic stroke showed significant gains in several clinical scales (36). A more recent controlled clinical trial in chronic stroke showed greater gains after Amadeo training than dose-matched conventional therapy, along with normalizing some aspects of interhemispheric connectivity after robot training (37). The Hand-of-Hope is an EMG-controlled exoskeleton with linkages that couple joints within each digit, decreasing the number of needed actuators. Clinical trials with this robot have shown significant impairment reductions and functional gains in chronic stroke subjects (38, 39). The FINGER robot provides assistance to the index and middle fingers as the subject plays a video game that simulates playing a guitar. A clinical trial reported significant gains in several clinical scales, with authors noting that subjects with impaired proprioception benefited less from the training (40).

Previously, our lab developed a Hand Exoskeleton Rehabilitation Robot (HEXORR I) (41) to retrain hand control and function. HEXORR I is a tabletop exoskeleton device that allows practice of finger and thumb movement integrated with video games. Compared to other hand robots, HEXORR I is unique in the use of a tone-compensation algorithm that measures the resistance to passive extension movement and applies extension assistance to counter this resistance (42). An additional novelty is the auto-adaptation algorithm that alters the shape and magnitude of the assistance profile to achieve a desired target performance level. In theory with this approach, the patient still has control of initiation, maintenance and termination of movement, but does not have to overcome the resistance from increased flexor tone during extension movement. In an initial pilot study, nine chronic stroke subjects showed significant improvements after 18 treatment sessions in range of motion, grip strength, and the hand component of the Fugl-Meyer score after HEXORR I use (43). Since then, HEXORR II was developed, which includes several hardware design changes to improve the performance of the robot, reduce the setup time and make the training sessions more engaging by implementation of a larger repertoire of games. In this study, we describe HEXORR II and report the results of a clinical trial that tested for a dosage effect from the robotic therapy. The hypothesis was that a significant dosage effect would be present when comparing 12 and 24 sessions of HEXORR II training, and that gains in finger extension ability and upper extremity function would be significant post treatment and at a 6 month followup.

## Methods

### Study Design

All testing protocols were approved by the MedStar Health Research Institute human subjects institutional review board (protocol 2012-315) and all subjects provided written consent. The trial was registered with clinicaltrials.gov (NCT04536987), where the underlying data will be made available. The inclusion criteria were: (1) a diagnosis of stroke more than 6 months prior to randomization; (2) presence of voluntary hand activity indicated by a score of at least 1 on the finger mass extension portion of the Fugl-Meyer Test of Motor Function (44), indicating the ability to release a mass flexion grip; (3) adequate cognitive status, as determined by Mini-Mental Status Examination (45) score > 24. Subjects were excluded if they: (1) were under the influence of antispasticity medications during the study; (2) had MCP and IP passive extension limit > 30° from full extension (to exclude patients with significant contracture); (3) had pain that interfered with daily activities; (4) had excessive tone in the fingers and thumb as determined by Ashworth (46) scores ≥ 3; (5) had severe sensory loss or hemispatial neglect as determined by a neurological clinical exam; (6) had any other medical conditions that affected their upper extremity function or their ability to complete the study protocols.

**Figure 1:**
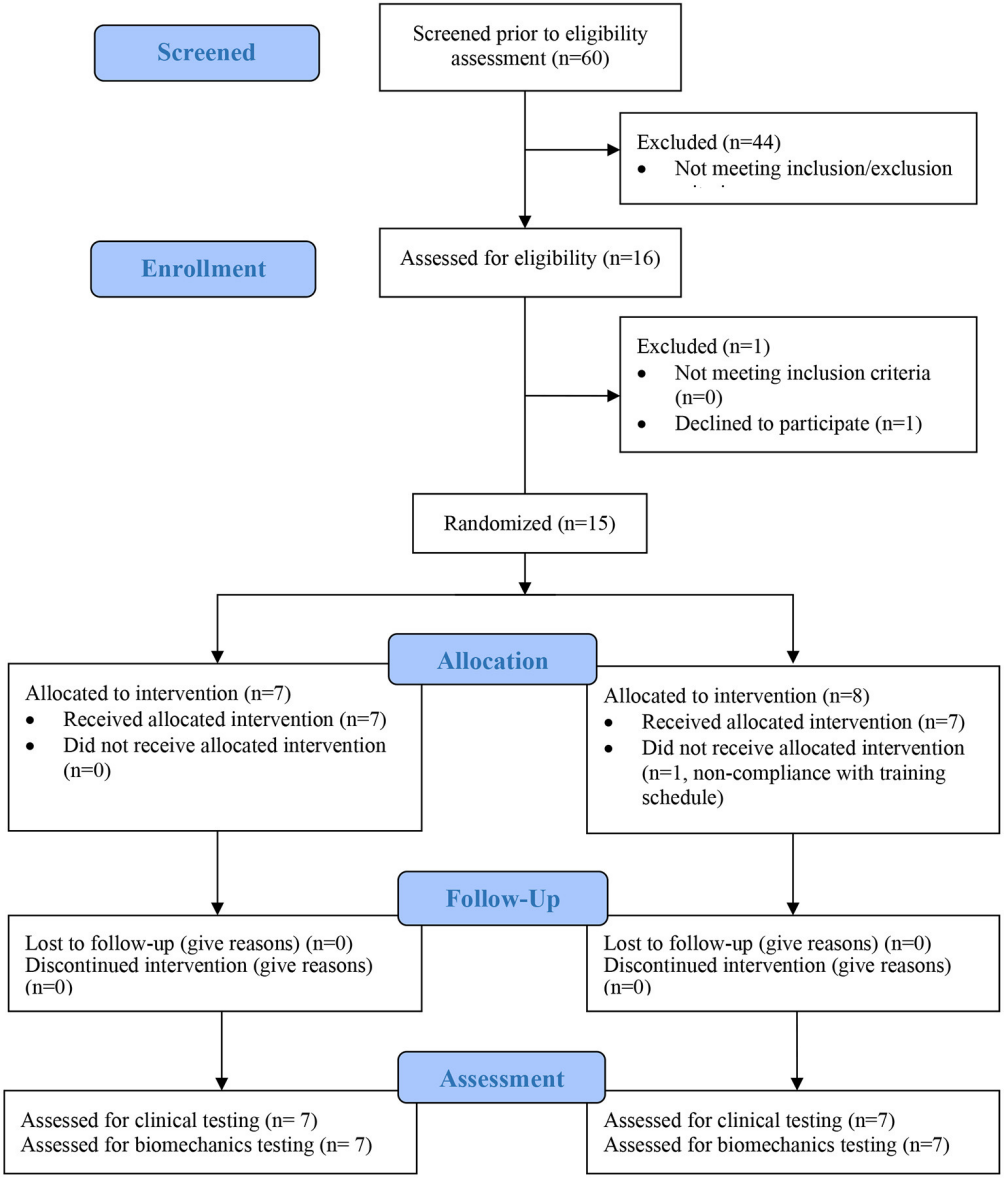
CONSORT flow diagram.

**Figure 2:**
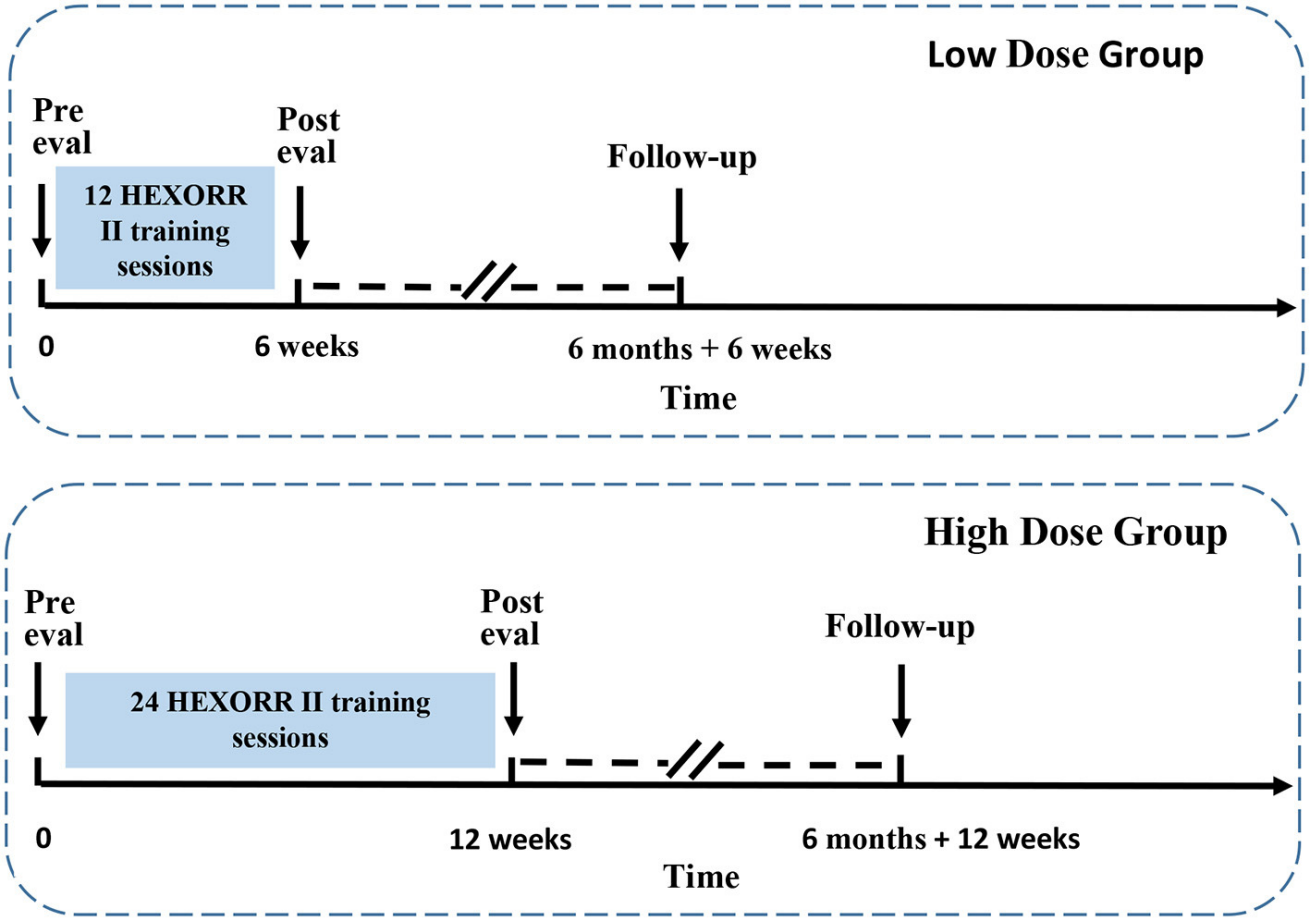
Description of timelines of HEXORR II training and evaluation. Top graph shows the training duration and evaluation time points of low dose group. Bottom graph shows the training duration and evaluation time points of high dose group. Two dashed lines, along with two slash lines indicate a portion of 6-month period are removed from the graph for clarity.

Each subject was randomized to either 12 or 24 training sessions of 1.5 h each. Subjects received two sessions per week, so the training duration was 6 and 12 weeks, respectively. In each session, the subjects received robotic therapy supervised by a technician. The subjects also completed pre-training, a post-raining, and 6 month follow-up evaluation sessions involving clinical scales and biomechanical motion capture. Figure 1 shows the CONSORT flow chart for the study. Subjects were aware that the study design entailed random assignment to high and low dosage groups. The staff providing therapy and the biomechanical evaluations were aware of the group assignment of all subjects. The person performing the clinical evaluations was unaware of the study design. Figure 2 shows the treatment and evaluation sequence for the two groups.

### Power Analysis

In a previously published HEXORR I study (43), 6 chronic stroke subjects received a higher dosage (more than 18 sessions) and showed increased gains of 2.17 pts in the FM hand component, whereas 9 chronic stroke subjects only received 18 treatment sessions and had gains in FM hand component of 1.86 points. This data was used to estimate a priori sample size for this study. The main hypothesis is a difference between low and high-dosage groups in the FM across the three time points, tested by repeated measures ANOVA. A correlation of coefficient of 0.85 is assumed between the dependent means (FM-UE score) within subject across all time points to be tested. All outcome measures evaluated at post training and follow-up time points will be expected to improve in comparison to the pre training evaluation. Outcome measures evaluated at post training for participants who completed 24 sessions will be expected to be further improved, in comparison of those who just completed 12 sessions. Thus, we performed a one-tail power analysis for comparisons of dependent means (matched pairs) with type I error probability of 5% (α = 0.05). The analysis resulted in a required minimum sample size of seven participants per group to attain a power of 80% for detecting a difference between 12 sessions vs. 24 sessions of treatment.

### Hand Exoskeleton Rehabilitation Robot (HEXORR II) (2nd Generation)

**Figure 3:**
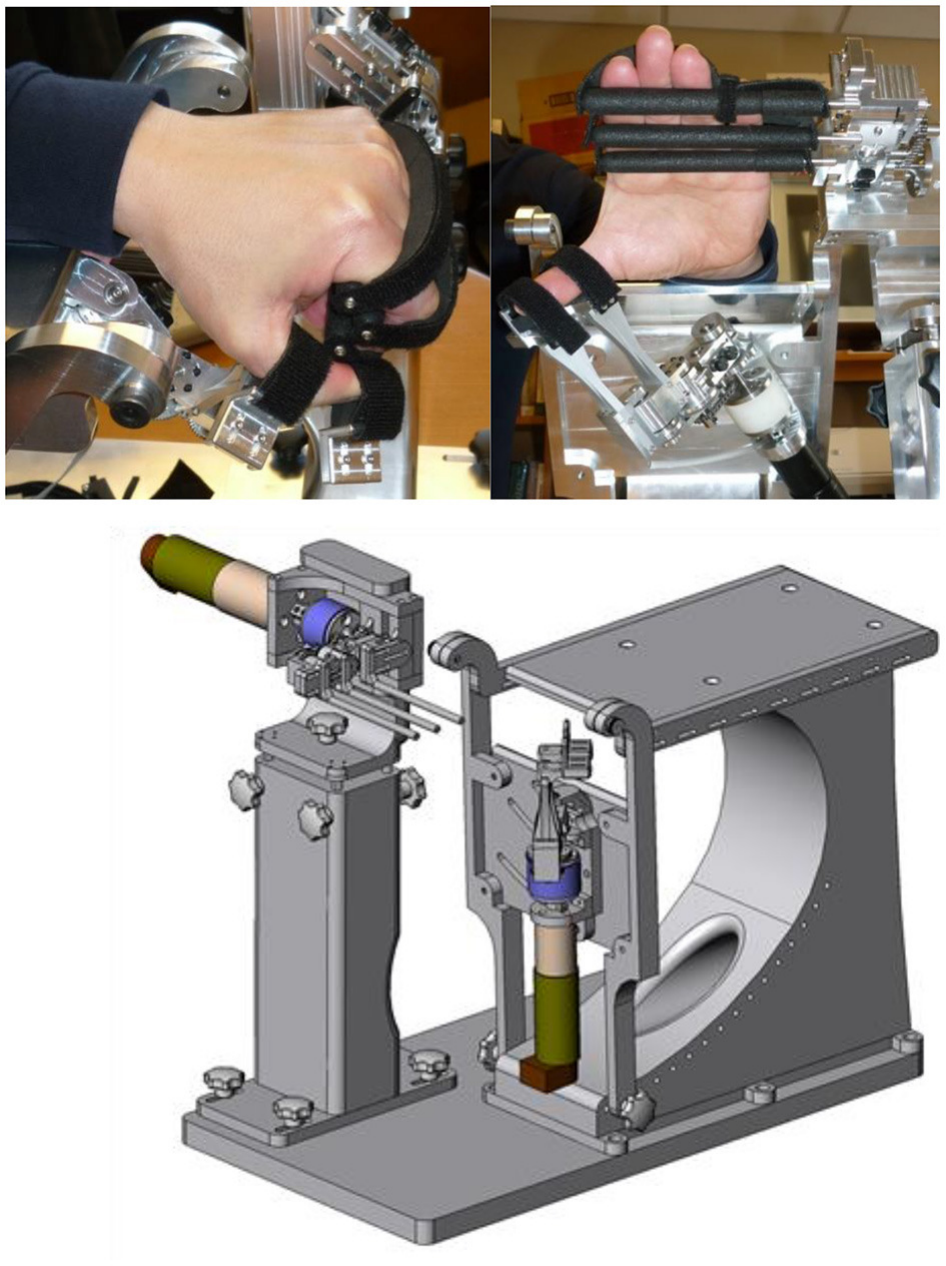
Pictures of the HEXORR II. The thumb flexion/extension plane can be adjusted by rotating the thumb actuator about two independent axes through the thumb CMC and locked in place.

HEXORR II maintains the basic functionality of the first-generation device (41, 43), but the mechanism has been completely redesigned to improve usability and performance. A single motor (Maxon RE40, GP42C 26/1, 4.4 Nm peak continuous torque) aligned with the metacarpophalangeal (MCP) of the fingers, assists synchronous movement of the four fingers (Figure 3). The motor drives a three-link serial linkage with three joints coupled together mechanically with a chain and gear mechanism, so that it can be controlled with a single motor. The link lengths of the serial linkage are adjustable so that the three joints can be aligned as close as possible to the finger MCP, proximal interphalangeal (PIP) and distal interphalangeal (DIP) joints. The three links are each connected to a bar that applies forces to the palmar surfaces of the fingers, helping to keep the fingers in natural postures during flexion and extension movements. The bars extend into a single plane for full extension, and collapse into a small space for full flexion. The thumb is controlled by a second motor (Maxon RE32, GP32C 33/1, 3.2 Nm peak continuous torque) that drives a similar linkage that has joints aligned with the thumb carpometacarpal (CMC), MCP and interphalangeal (IP) joints. Two pads that are strapped to the distal and proximal phalange of the thumb and move the thumb in flexion/extension in a single plane that can be adjusted for comfort. The forearm is strapped onto a horizontal surface. The horizontal surface extends across the wrist joint to support the palm of the hand, thereby restricting wrist flexion. A Matlab Simulink program (xPCtarget, Stateflow) controls the motors and provides feedback during training.

When compared to HEXORR I, the HEXORR II provided several additional capabilities. HEXORR I actuated the MCP and PIP finger joints, while HEXORR II actuated these joints and the DIP joints. In HEXORR II, there was a marked reduction in the time required to position and strap the hand into the device. Also the moving inertia and friction of the robot was reduced compared to HEXORR I, allowing more natural movement trajectories and less resistance to free movement. The training software was improved with the addition of several video games that were not available previously. Both devices incorporated the same auto-adaptation algorithm for adjusting the assistance level, but the new software also provided a user interface for the experimenter to manually adjust the assistance profile as needed.

### Training Protocol

For the first session, the hand is placed in HEXORR II and three slow passive stretches of the fingers are performed. The movement is constant velocity and very slow (10°/s). We retained the motor torque applied during these stretches as the starting point for the torque vs. angle extension assistance profiles provided during training. The subjects then spent the rest of the session playing several different types of games, while assisted by the robot.

The primary therapy mode game was the Gate Game. It required the subject to extend and flex the fingers and thumb to guide two balls through two openings in a gate that sweeps across the screen. If the digits were not opened in time to pass the balls through the gate, the digits were moved by the robot to full extension, before the next flexion movement was prompted. An adaptation algorithm was implemented where the target performance was achievement of 2 of 3 consecutive gates. If 2 of 3 gates were successfully completed, the assistance profile was kept unchanged. If the performance was below this level, the assistance was increased by 0.1 Nm over the range from the peak extension angle achieved in the prior three trials to full extension. If the performance was perfect over three trials, the assistance profile was scaled down by a 10%. This adaptation strategy allowed for the shape of the torque vs. angle profile to evolve as well as the overall amplitude (43). Subjects performed 3 blocks of 30 gates in each session.

The remainder of the 90 min session was spent playing secondary video games. The subject could select from four different PC games, which were played by moving the thumb and fingers in HEXORR II with assistance. These games included three PC commercial games and one custom designed game. All these games had scoring systems and offered easy ways to set the game difficulty and to track individual's performance. All of the games were normally controlled by mouse movement. Interface electronics (Arduino) received input of finger and thumb angles from the Matlab robot controller (RS232 communication protocol) and mouse emulator code on the Arduino controlled the PC mouse position on the computer screen through the USB port of the PC. For games that required a mouse click, a push button was controlled by the unaffected hand and provided input to one of the Arduino digital ports and integrated into the mouse control. In this way, no modification of the commercially available PC games was needed and an array of games could be integrated into the training. The most up to date assistance profile was used in this mode, but was not automatically adapted as in the Gate Game. Games included Angry Birds, Bubble Shooter, Shopping and Ping-Pong. Studies show gamification of upper limb stroke therapy increases compliance and motivation (47–49).

### Evaluation Sessions

Each evaluation session consisted of clinical measures and biomechanical measures. The clinical measures included the Action Research Arm Test (ARAT) (50) for grasp, grip, pinch, and gross arm movement; the Fugl-Meyer (FM) for motor impairment; the Modified Ashworth scale for hypertonia at the fingers, wrist and elbow; the Motor Activity Log (MAL) (51) to assess use of the impaired limb in ADL. The MAL amount of use score was retained for statistical analysis. A Jamar dynamometer (JAMAR 5030J1 Hand Dynamometer) was used to measure grip strength at each time point.

**Figure 4:**
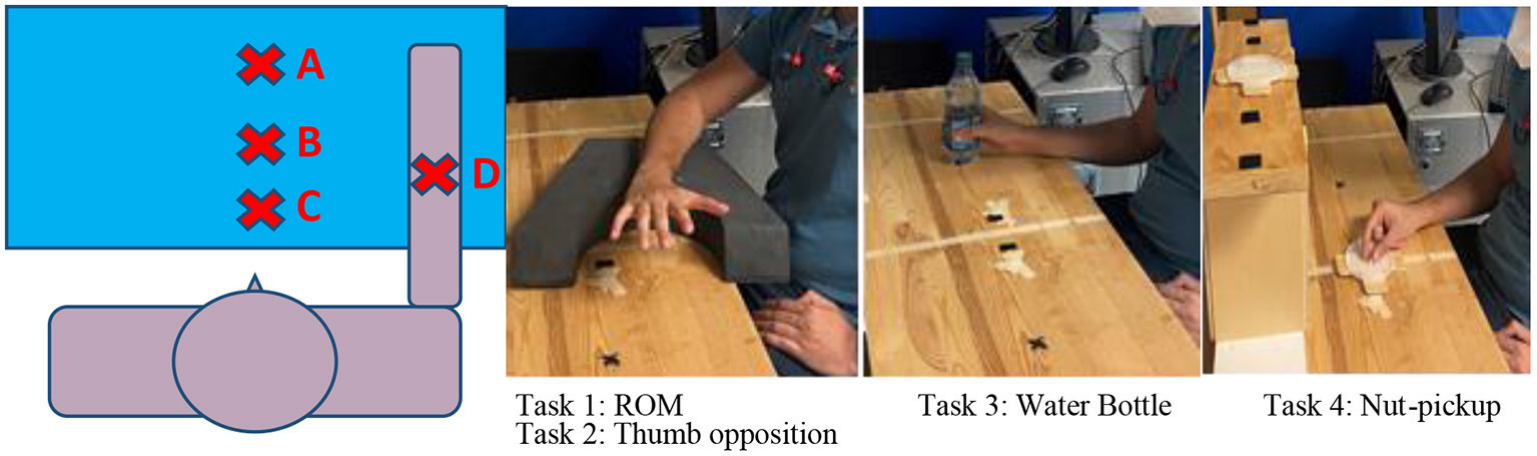
Subjects were seated 4” from the testing table, measured from their diaphragm to the table edge. The subject was assisted to outstretch their arm anteriorly to 90° shoulder flexion with elbow extended. Mark A was placed on the table in the center of the hand in this outstretched position. Mark B was 11” anterior to the subject's diagram and mark C was 6” directly in front of the subject's diaphragm. Mark D was 8” anterior to the subject's diagram. Task #1 (Digit ROM) and Task #2 (Thumb opposition) were performed with the hand at mark C. Task #3 (Water Bottle) was performed with the hand starting and ending at mark C, and the bottle at mark D. Task #4 (Nut pickup) also started with the hand at mark C, with the nut placed at mark B, and the target drop location on a shoulder height shelf at mark A.

For the biomechanical measures, subjects were seated in front of a table at a standardized position and four tasks were performed. Figure 4 shows the layout for the testing and the locations of the task objects. The tasks were: Task (1) Full digit ROM: straightening the fingers as much as possible from a closed fist position, with the hand in a pronated position at midline and the forearm supported against gravity; Task (2) Thumb Opposition: touching the thumb to the tip of the 5th digit, to test for thumb abduction range of motion; Task (3) Water Bottle: grasp a water bottle placed lateral to a standard starting point at midline and bring this water bottle to mouth to drink; Task (4) Nut pickup: pick up a small nut placed at midline and put it on the top of a shelf. Each task was done twice, and each trial was 40 s. Metrics from each trial were averaged across the two trials of each task before statistical analysis.

The kinematics were measured using an electromagnetic tracker (MiniBirds®, Ascension Technologies). Sensors were taped to nail of the thumb, index, middle and ring fingers. Sensors are also taped to the dorsum of the hand and forearm. An additional sensor is taped to the C7 vertebrae. Using commercially available biomechanics software (MotionMonitor, Innsport Inc.), anatomical landmarks were digitized and segment coordinate frames calculated for the hand, forearm and trunk. Raw data were exported into a custom Matlab program that calculated several metrics. For the finger markers, the total flexion angle was calculated for the finger distal phalange relative to the hand segment. This represents the sum of flexion from all three joints of each digit. For the thumb, the abduction and flexion angles were both calculated. Standard Euler sequences were used for these calculations (52). Finger extension deficit was calculated as the smallest flexion angle (largest extension angle) achieved during the trial, averaged across the four digits measured. Finger range of motion (ROM) was calculated as the difference between the largest and smallest flexion angle achieved, averaged across the four digits. Trunk ROM was determined by calculating the farthest movement of the trunk coordinate frame relative to the starting point at the beginning of the trial, in forward and lateral directions. Hand displacement (due to proximal arm movement during the reaching tasks) was calculated similarly, except trunk movement in each direction was subtracted from hand movement first, so that the hand displacement metric was associated with arm movement only. We included hand displacement because of the possibility that improved hand function would lead to increased use of the upper extremity outside of the therapy provided.

### Data Analysis

For each outcome, a repeated measures ANOVA was used with between subjects factor of group (12 sessions or 24 sessions) and within subjects factor of time (pre, post, followup). Simple within-subject preplanned contrasts were performed following the ANOVA to detect changes from pre to post, and from pre to followup. We tested that all dependent variables met the assumptions for parametric statistics. Normality was tested with the Kolmogorov-Smirnov Test. Homogeneity of variance was tested with Levene's Test. Outliers were detected if the z score was >3. For dependent variables that did not meet these assumptions, between group differences were tested with Mann-Whitney *U*-Test, and the Wilcoxon Signed Ranks Test was used to test for significant changes between time points.

## Results

Fifteen subjects were enrolled in this study and 1 subject withdrew due to non-compliance with the training schedule. The remaining 14 subjects were randomized to the 12 session dosage (7 subjects) or the 24 session dosage (7 subjects). Table 1 reports the subject characteristics. The mean age was 62.3 (11.7) years, and the mean time since stroke was 28.7 (18.7) months. Seven males and seven females completed the study, and the right limb was more affected in eight subjects. At baseline, the mean Fugl-Meyer scores were 38.6 ± 13.9 in the low dose group and 29.3 ± 7.7 in the high-dose group. The baseline ARAT scores were 22.4 ± 21.6 in the low-dose group and 15.4 ± 12.7 in the high dose group. Mean age was 62.9 ± 12.7 in the low dose group and 63.7 ± 10.6 in the high dose group. Chronicity was 26.4 ± 18.6 months in the low dose group and 29.3 ± 18.4 months in the high dose group. The two dosage groups were not significantly different at baseline in the FM, ARAT, age or chronicity (*p* > 0.14).

**Table 1:** Participant Characteristics.

**Subject**	**Group assignment**	**Age (years old)**	**Sex**	**Stroke type**	**Affected side**	**chronicity (months)**	**Fugl-Meyer**	**ARAT**
1	Low dose	77	F	Ischemic	Right	9	56	48
2	Low dose	75	M	Ischemic	Right	14	34	12
3	Low dose	60	M	infarct and hemorrhage	Left	19	57	54
4	Low dose	64	F	Ischemic	Left	27	30	3
5	Low dose	39	M	Ischemic	Right	57	43	30
6	Low dose	67	F	Ischemic	Right	47	21	6
7	Low dose	58	M	Ischemic	Left	12	29	4
8	High dose	67	M	Ischemic	Left	45	40	31
9	High dose	44	F	hemorrhagic	Right	22	32	13
10	High dose	64	M	Ischemic	Left	28	33	33
11	High dose	75	F	Ischemic	Left	20	29	19
12	High dose	61	M	Ischemic	Left	63	15	3
13	High dose	60	F	Ischemic	Left	13	30	4
14	High dose	75	F	Ischemic	Right	14	26	5

*MCA, Middle Cerebral Artery; ARAT, Action Research Arm Test*.

**Figure 5:**
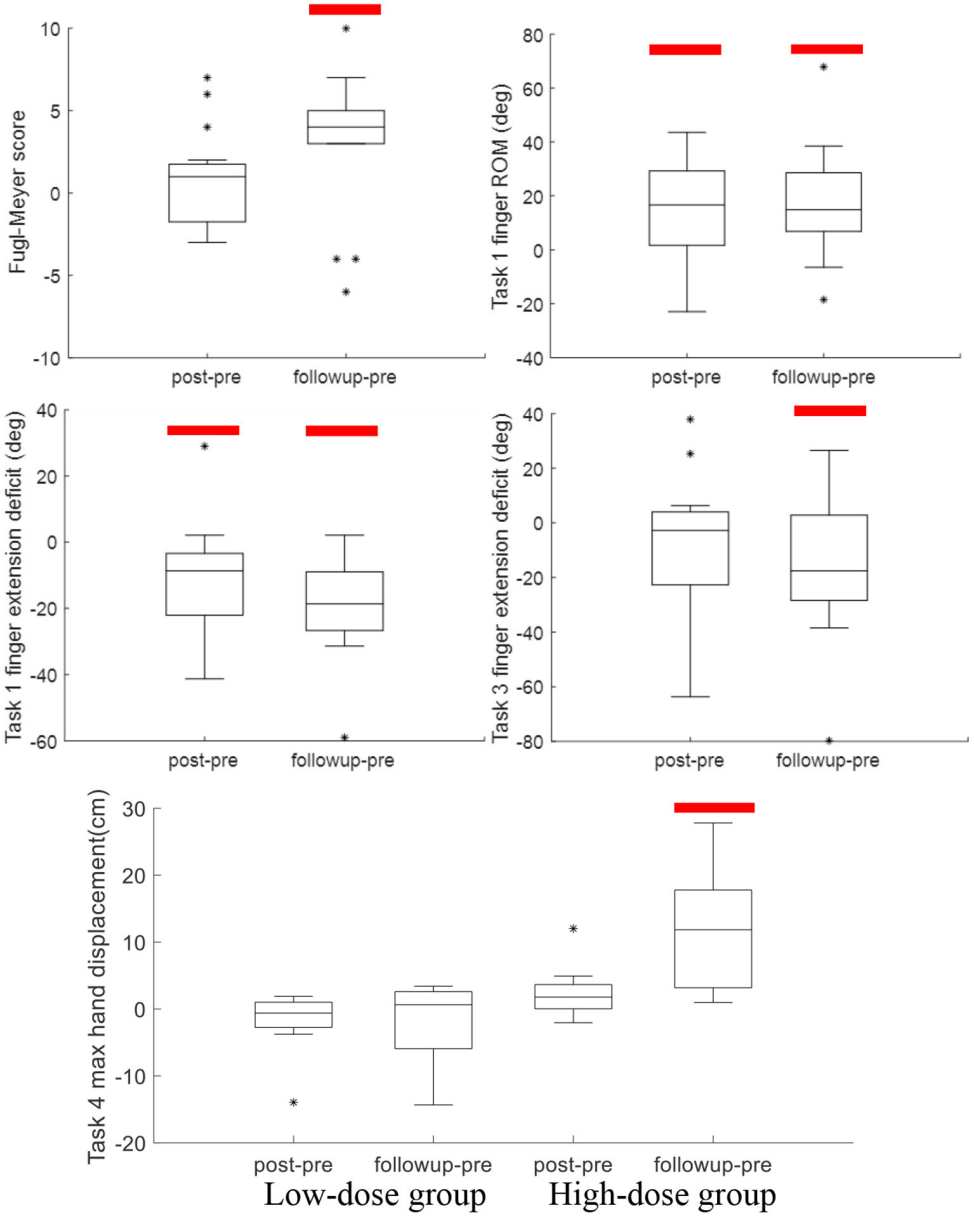
Boxplots of post-pre and followup-pre changes in outcome measures with significant changes, indicated by the red bar. Significant improvements in Ashworth scores (Table 2) are not shown.

Table 2 reports the statistical analysis of the clinical outcome measures. Fugl-Meyer scores increased over the three time points and RM-ANOVA reported a significant time factor (*p* = 0.045). At the 6 month followup, the Fugl-Meyer had increased 2.9 points relative to baseline (*p* = 0.033, Cohen's d = 0.24). Significant effects were also found in the Ashworth test. For the Ashworth test of finger flexors, the time factor was significant (*p* = 0.024), indicating change over time in finger flexor tone. Paired *t*-tests found a trend for decreased finger flexor tone at the follow up time point (*p* = 0.068, Cohen's d = 0.45). Results at the other joints were similar, with significant improvements in tone at follow up at the wrist (*p* = 0.031, Cohen's d = 0.50), pronators (*p* = 0.029, Cohen's d = 0.64) and elbow flexors (*p* = 0.028, Cohen's d = 0.49). Significant score changes are shown in Figure 5. There were no significant changes over time for the MAL, ARAT, Ashworth (extensors) or grip strength. The group and group^*^time factors were not significant for any of the clinical measures.

**Table 2:** Summary of clinical outcome data: mean(sd).

	**Pre**		**Post**		**Followup**		**Group** **(*p*)**	**Time** **(*p*)**	**Time × group** **(*p*)**	**Post-pre** **(*p*)**	**Followup-pre (p)**
Fugl-Meyer	33.9	(11.8)	34.7	(10.5)	36.8	(12.3)	0.165	0.045	0.546	0.374	0.033
ARAT	18.9	(17.4)	19.0	(15.8)	19.3	(16.1)	0.485	0.884	0.576	0.965	0.849
Motor activity log	1.54	(1.38)	1.63	(1.30)	1.55	(1.21)	0.169	0.912	0.454	0.712	0.958
Ash-finger flexors	1.18	(0.89)	1.25	(0.78)	0.79	(0.85)	0.739	0.024	0.329	0.635	0.068
Ash-wrist flexors	1.04	(1.03)	0.96	(0.97)	0.57	(0.81)	0.466	0.104	0.804	0.745	0.031
Ash-pronators	1.18	(1.01)	1.07	(0.98)	0.61	(0.76)	0.591	0.027	0.067	0.551	0.029
Ash-elbow flexors	1.11	(0.90)	1.00	(0.92)	0.71	(0.70)	0.394	0.103	0.381	0.568	0.028
Grip strength (lbs)	19.2	(15.4)	24.5	(22.4)	21.5	(15.6)	0.470	0.237	0.258	0.050	0.596

*Ash-modified ashworth scale*.

**Figure 6:**
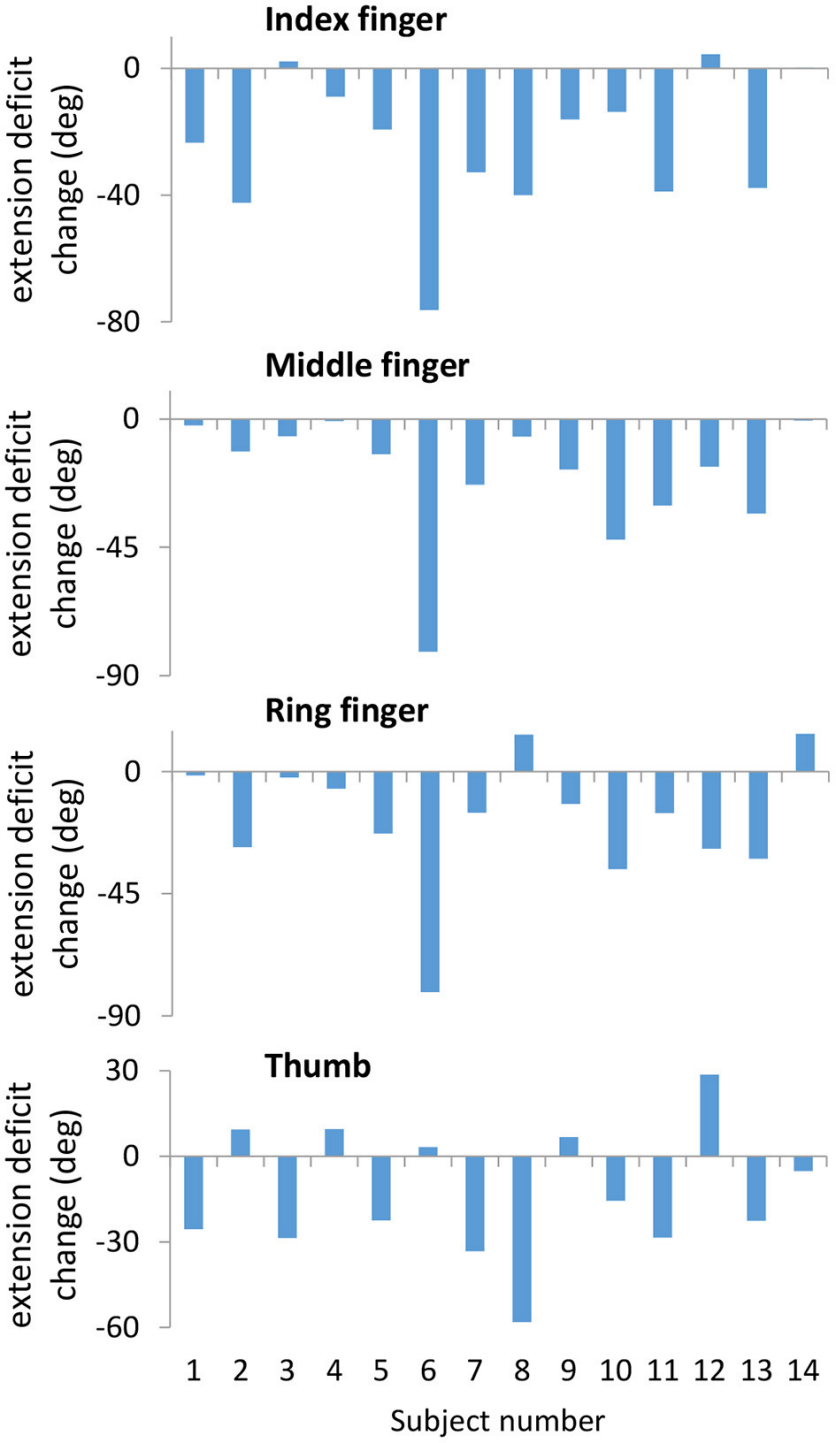
Decreased extension deficit at the follow-up time point compared to the pre-training time point measured during Task 1. Changes are strongly correlated across the Index, Middle, and Ring fingers. Subject numbers correspond to subject numbers in Table 1.

Table 3 reports the results of RM-ANOVA for the kinematic variables. For Task 1, closing and opening the hand with the forearm supported against gravity, RM-ANOVA reported that finger and thumb movements showed increased range of motion (*p* = 0.028) and finger extension ability (*p* = 0.006) over the three time points. Relative to baseline, finger extension increased (gain = 11.9 ± 18.3°, p = 0.030, Cohen's d = 0.22) immediately after training and at the 6-month followup (gain = 19.0 ± 15.7°, *p* < 0.001, Cohen's d = 0.36). Finger extension gains across all 14 subjects at the followup time point are shown in Figure 6. Range of motion increased in parallel; the increase was significant at the post timepoint (gain = 12.7 ± 21.1°, *p* = 0.042, Cohen's d = 0.23) and at the 6-month followup (gain = 17.5 ± 21.3°, *p* = 0.009, Cohen's d = 0.30). In Task 3, where the subject must reach out and grasp a water bottle, finger extension deficit did decrease from pre-training (56.6 ± 40.2°) over the three time points to the followup (40.7 ± 32.4°), but this improvement in finger extension did not reach statistical significance (time factor in RM-ANOVA, *p* = 0.057). However planned contrasts at the post and followup timepoints found significantly greater finger extension in Task 3 at followup (*p* = 0.044, Cohen's d = 0.44).

**Table 3:** Summary of biomechanics data: mean(sd).

**Metric**	**Pre**		**Post**		**Followup**		**Group** **(*p*)**	**Time** **(*p*)**	Time*group **(*p*)**	**Post-pre** **(*p*)**	**Followup-pre (*p*)**
**TASK 1**											
finger extension deficit (deg)	73.1	(53.9)	61.2	(53.5)	54.1	(52.2)	0.952	0.006	0.894	0.030	<0.001
finger ROM (deg)	97.7	(55.4)	110.4	(56.7)	115.2	(63.2)	0.718	0.028	0.544	0.042	0.009
**TASK 2**											
thumb abduction max (deg)	39.6	(16.2)	40.6	(9.8)	42.9	(16.8)	0.413	0.689	0.608	0.808	0.607
**TASK 3**											
finger extension deficit (deg)	56.6	(40.2)	48.6	(33.6)	40.7	(32.4)	0.804	0.057	0.345	0.263	0.044
finger ROM (deg)	79.1	(38.7)	79.9	(20.7)	78.9	(32.9)	0.276	0.989	0.433	0.916	0.982
hand displacement max (cm)	30.3	(9.0)	33.3	(15.4)	28.9	(12.8)	0.261	0.358	0.618	0.278	0.564
trunk forward disp. max (cm)	10.1	(6.2)	11.0	(5.6)	9.6	(5.8)	0.596	0.573	0.199	0.482	0.760
trunk lateral disp. max (cm)	11.8	(12.4)	8.2	(4.4)	9.2	(4.5)	>.71^#^	n/a	n/a	0.594^*^	0.875^*^
**TASK 4**											
hand displacement max (cm)	24.8	(16.0)	25.0	(14.9)	29.5	(15.2)	0.497	0.037	0.004	0.888	0.135
trunk forward disp. max (cm)	10.4	(5.8)	10.3	(4.3)	9.9	(5.2)	0.162	0.914	0.142	0.931	0.733
trunk lateral disp. max (cm)	8.7	(7.2)	7.0	(2.7)	7.2	(2.8)	>0.53^#^	n/a	n/a	0.778^*^	0.778^*^

* *Wilcoxon Signed Ranks Test*.

# *Mann-Whitney U-Test*.

In Task 4, picking up a nut and placing on a high shelf, max hand displacement increased over time (*p* = 0.037) and also there was a group x time interaction (*p* = 0.004). This was due to no increase in the low-dose subjects (*p* = 0.539) and a significant increase in the high-dose group (*p* = 0.005). Compared to baseline, the high-dose group increased hand displacement by 11.8 ± 0.10 cm at the followup timepoint (*p* = 0.020, Cohen's d = 0.78). There were no other significant effects in the kinematic variables (Table 3).

## Discussion

HEXORR II therapy produced reductions in upper extremity impairments, as measured by significant gains in the Fugl-Meyer score at the 6 month followup. The largest changes were at the 6-month time point and included a significant reduction of flexor tone, increased finger ROM, and decreased finger extension deficit. The improvement in finger extension is noteworthy, as we are aware of only one prior study of hand robotics reporting an increase of extension range that was retained 6 months after the intervention (35). However, there were no changes on a measure of upper extremity function (ARAT). No dosage effects were found, with the exception of increased hand displacement during the task requiring forward reach (Task 4) in the high-dose group, and no change in the low-dose group.

We observed significant long-term reductions in hypertonia at the followup, as measured by the Modified Ashworth Scale. To our knowledge, this is a novel result not previously reported for hand robotic devices. This reduction in flexor tone may have contributed to the increased finger extension ability also reported at the followup time point. While the passive stretching performed at the beginning of each session was limited to only a few repetitions, we applied a stretch and hold movement immediately after each active extension attempt during the Gate game. The possibility of co-contraction of flexors during this stretch would have led to eccentric contraction of flexors, which may decrease hypertonia following neurologic injury (53). Studies with the X-Glove have shown that a 30 min period of cyclic passive stretching can transiently improve active motor performance in stroke patients, with effects carrying over across sessions in subacute stroke (54). Improvements in subacute stroke subjects were reported in measures of impairment and function following training that included 30 min of passive cyclic stretching followed by active-assisted, task practice (34). Authors attributed the passive stretching to facilitating the effectiveness of the active training and preventing any increases in spasticity. There is also some evidence that orthotic-based static stretching can decrease upper extremity spasticity, although there is no evidence this alone will improve motor performance (55, 56). Thus, our results contribute to the evidence supporting further study into the use of robotics to integrate stretching protocols into active motor retraining.

One unique aspects of this study was the detailed biomechanical analysis that reported the kinematics of finger and arm movements under several conditions. Results support the use of HEXORR II in combination with practice of functional upper extremity tasks. The HEXORR II focuses on hand movement with the forearm and wrist immobilized and the arm supported against gravity. Hand movements in conjunction with proximal arm movements were not practiced, as is required for functional use of the upper extremity. This might explain the gains in an impairment scale (Fugl-Meyer), but no gains in a test of function that tests the ability to pick up and place objects (ARAT). There is strong evidence that control of the fingers degrades when proximal muscles must support the arm against gravity (57–59). These studies are consistent with our kinematic results, as finger extension did improve significantly when tested with the arm supported against gravity, but finger extension during reach and grasp tasks did not improve significantly at the post time point (however finger extension gains in the water bottle task did reach significance at followup). Thus, the training of distal hand control did produce gains in the training task, but did not generalize strongly to improved function in reach and grasp tasks without additional practice of unsupported reach and grasp tasks. Two large multisite clinical trials of whole arm and hand robotic training also found similar results. The Armin was found to produce greater gains in the Fugl-Meyer scale than conventional therapy, but had no advantages in a motor function scale (60). In the RATULS study, robotic therapy produced greater gains in the Fugl-Meyer compared to usual and customary care, but had no advantage on the ARAT (61). In contrast, studies which combined robotic hand training with functional task practice have reported gains in functional scales. A recent study with Amadeo reported gains in the nine-hole Peg test, when subjects received the robotic training after a 3 h session of physiotherapy that included 45 min of occupational therapy and 45 min of biomechanical training of upper and lower limbs (37). Several other studies have used wearable hand robots [X-Glove (34), VAEDA (35), Hand-of-Hope (38, 39), HandSOME (21)] that enabled practice of reach, grasp and release tasks with robotic assistance to hand movement. All of these studies reported significant gains on a variety of functional scales. Thus, functional gains with devices similar to HEXORR II that focus on distal control only, might be achieved by integrating practice of coordinated proximal and distal limb control, as is often done during conventional therapy. Robotic and conventional therapies promote distinct patterns of motor recovery (62), and there is evidence from clinical trials that the addition of conventional task practice to robotic therapy is superior to robotic therapy alone (63–65).

Our results are generally consistent with prior studies of robots that train the fingers in isolation from the proximal arm. We found a 2.9 point change in the Fugl-Meyer at followup, while therapy using the FINGER robot reported gains of 1.8-3.7 at followup (40), and a study using the Amadeo robot reported a 5.1 Fugl-Meyer point change (36). Our previous clinical study using HEXORR I also reported an increase in finger extension ability and significant gains in the Fugl-Meyer hand section subscore after 18 h of training (43). However our prior study also reported grip strength increases and significant gains in the ARAT in a subgroup of low tone subjects. The group average Fugl-Meyer gain of 2.9 points is lower than the minimally clinically important difference (MCID) values reported by other research groups. Page et al. (66) reported that the estimated MCID of the UE-FM score is as low as 4.25 points whereas Greisberger et al. (67) used an MCID of 5.2 points for the UE-FM. However, if we use five points as MCID on the UE-FM, five subjects achieved MCID at the followup (gains of 5,5,5,7,10 points). If we use 4 points as MCID, a total of 9 subjects achieved MCID at the followup (4 subjects had gains of 4 points at followup).

Our current study did not find any changes in the ARAT or grip strength. This might be explained by fact that the prior HEXORR I training included a squeezing task that required generation of targeted isometric matching flexion forces from the fingers and thumb, followed by releasing of the grip within a certain time interval. This squeeze and release practice might have helped subjects improve grip force and the ARAT, which involves grasping and releasing objects. We elected to drop the squeezing task from the current study to increase the number of repetitions that focused on extension movement. The prior studies with the FINGER and Amadeo also reported gains in functional scales (ARAT, Box-and-Blocks, Jebsen Taylor Hand Function Test), while we did not see any improvement on functional scales in this study. One possible explanation is the low functional level of our subjects. Our mean intake Fugl-Meyer score was lower than these other two studies, and our intake ARAT scores were low (mean of 19/57 points), with 6 of our subjects having an intake ARAT of 6 points or less. In more severely impaired subjects, practice of grip or squeezing tasks might be important to include with finger extension training. Gains in biomechanical and clinical measures were largest at the followup time point, with some metrics even showing non-significant gains immediately after training, but significant improvements at the 6-month time point (Fugl-Meyer, Ashworth, Task 3 finger extension). A similar result was reported by Thielbar et al., who found that gains in grip aperture were not significant after robotic hand training, but continued to increase during the 10 week followup and became significant (35). According to the “threshold” hypothesis (68), initial gains at the post-treatment time point could lead to further improvements during the followup period if spontaneous movement of the affected limb increased. However, MAL scores did not indicate increased use of the more-affected arm within ADL tasks. Future studies may consider using objective methods to assess upper extremity activity as an outcome measure (69).

This study randomly assigned participants to either 12 sessions or 24 sessions. Based on RM-ANONA analysis, the group^*^time factors were not significant for our clinical outcomes measures (Fugl-Meyer and ARAT). The only significant between-group difference was increase in hand displacement in the high dose group during a forward reaching task that appeared at followup, which suggests practice of reaching tasks during this period. It should be noted that the treatment period in the high dose group was 3 months, very long compared to other studies. Long duration training periods might be needed to affect durable change in functional reach and grasp tasks in chronic stroke patients. The low number of subjects in the treatment groups in our study limited our ability to detect a dosage effect; however, a larger scale clinical trial also did not report dosage effects (70). More study is needed to understand why a dosage effect is often not present in neuro-rehabilitation clinical trials of the upper extremity.

### Limitations

This study has several limitations that should be noted. We powered our study using data from the FM-UE measure, and we cannot rule out the possibility that our null result for ARAT, MAL, and some of the biomechanical measures were due to lack of power, with too small of a sample for comparison of dosage effects (71). The average of two trials for kinematic analysis is relatively lower than the recommended number of trials for accurate assessment of upper limb movement after stroke (72, 73). An additional measurement time point at 6 weeks in the high-dose group would have provided more information on dose-response. The MCID used for FMA-UE in this study is considerably lower than the recent study in acute stroke individuals done by Hiragami et. al (74), whereas MCID used for ARAT in this study is considerably lower than another acute stroke study done by Lang et. al (75). HEXORR II allows practice of isolated thumb movement, but the other 4 fingers are coupled together. Inability to isolate these four fingers may have limited the therapy's effectiveness to target weak fingers, since a weak finger can be carried along by the actions of the other 3. Another limitation was that the automatic adaptation algorithm only operated during the Gate Game, and not the secondary games, which were commercially available PC games chosen for their professional graphics and potential to entertain the subject. However, the downside of this approach is that the robot controller has no knowledge of current performance during the game, so automatic adaptation of assistance was not possible. The device currently is not portable, but getting into the device was straightforward and the potential for a home based portable device that can be used independently by patients seems possible if the overall size and footprint of the device can be reduced.

## Conclusions

Overall, HEXORR II training reduced impairment levels, increased finger extension ability and decreased flexor hypertonia at the 6-month followup. Future work with HEXORR II should focus on integrating it with functional task practice and incorporating grip and squeezing tasks. Notably, the easy setup and gaming interface make HEXORR II a potential home therapy device that could be used in conjunction with outpatient therapy, where they would receive functional upper extremity task practice.

## Data Availability Statement

The original contributions presented in the study are included in the article/supplementary material, further inquiries can be directed to the corresponding author/s.

## Ethics Statement

The studies involving human participants were reviewed and approved by Human Research Protection Program at Medstar Health Research Institute. The patients/participants provided their written informed consent to participate in this study.

## Author Contributions

JC assisted with the software development, data collection, interpretation of results, and was a major contributor to writing the manuscript. IB designed the mechanical aspects of the robotic device. DN performed subject recruitment, data collection, and assisted with interpretation of results. TC assisted with the mechanical design, assembly of the device, and data collection. MS and RC assisted with data collection and writing of the manuscript. PL conceived of the work, designed the clinical trial, assisted with the mechanical design of the robotic device, assisted with software development, performed data analysis, assisted with interpretation of results, and was a major contributor to writing the manuscript. All authors read and approved the final manuscript.

## Funding

This work was supported by the National Institutes of Health under grant R15 HD075166.

## Conflict of Interest

The authors declare that the research was conducted in the absence of any commercial or financial relationships that could be construed as a potential conflict of interest.

## Publisher's Note

All claims expressed in this article are solely those of the authors and do not necessarily represent those of their affiliated organizations, or those of the publisher, the editors and the reviewers. Any product that may be evaluated in this article, or claim that may be made by its manufacturer, is not guaranteed or endorsed by the publisher.
